# Effect of mother’s education on child’s nutritional status in the slums of Nairobi

**DOI:** 10.1186/1471-2431-12-80

**Published:** 2012-06-21

**Authors:** Benta A Abuya, James Ciera, Elizabeth Kimani-Murage

**Affiliations:** 1 Education Research Program, African Population & Health Research Center (APHRC), Kirawa Road, Off Peponi Road, P.O. Box 10787, 00100, Nairobi, Kenya; 2 African Institute for Development Policy (AFIDEP), Suite #29, Royal Offices, Mogotio Road, off Chiromo Lane, P.O. Box 14688, 00800, Westland’s, Nairobi, Kenya; 3 Health Systems and Challenges, African Population & Health Research Center (APHRC), Kirawa Road, Off Peponi Road, P.O. Box 10787, 00100, Nairobi, Kenya

**Keywords:** Education, Child stunting, Health, Urban slum, Kenya

## Abstract

**Background:**

Malnutrition continues to be a critical public health problem in sub-Saharan Africa. For example, in East Africa, 48 % of children under-five are stunted while 36 % are underweight. Poor health and poor nutrition are now more a characteristic of children living in the urban areas than of children in the rural areas. This is because the protective mechanism offered by the urban advantage in the past; that is, the health benefits that historically accrued to residents of cities as compared to residents in rural settings is being eroded due to increasing proportion of urban residents living in slum settings. This study sought to determine effect of mother’s education on child nutritional status of children living in slum settings.

**Methods:**

Data are from a maternal and child health project nested within the Nairobi Urban Health and Demographic Surveillance System (NUHDSS). The study involves 5156 children aged 0–42 months. Data on nutritional status used were collected between October 2009 and January 2010. We used binomial and multiple logistic regression to estimate the effect of education in the univariable and multivariable models respectively.

**Results:**

Results show that close to 40 % of children in the study are stunted. Maternal education is a strong predictor of child stunting with some minimal attenuation of the association by other factors at maternal, household and community level. Other factors including at child level: child birth weight and gender; maternal level: marital status, parity, pregnancy intentions, and health seeking behaviour; and household level: social economic status are also independently significantly associated with stunting.

**Conclusion:**

Overall, mothers’ education persists as a strong predictor of child’s nutritional status in urban slum settings, even after controlling for other factors. Given that stunting is a strong predictor of human capital, emphasis on girl-child education may contribute to breaking the poverty cycle in urban poor settings.

## Background

Malnutrition, particularly stunting, is still a severe public health problem in Sub-Saharan Africa [1]. For example, about 35 % of preschoolers are stunted, while 29 % are underweight in Sub-Saharan Africa [2]. In addition, the Fifth Global Nutritional Report estimates that in East Africa 44 % of children under-five were stunted while 31 % were underweight in 2005 [3]. Malnutrition has both short term and long term adverse ramifications. In the short-term for the individual, it is associated with ill health and mortality [4]. In the long-term, it leads to impaired cognitive development, poorer educational achievement and economic productivity [5,6].

Rapid growth in urban population has been experienced in the entire world in recent decades. For instance, the population in cities in the developing countries was 2 billion in 2000 and is expected to rise to about 4 billion in 2030 [7]. This rapid and uncontrolled urbanization in many Sub-Saharan African countries, Kenya included has resulted in a vast majority of people in urban areas living in slum conditions. The slums are characterized by overcrowding, filth, inadequate water supply, poor drainage, and poverty; leading to poor health for the urban dwellers [8,9]. According to Gould (1998), the effects of the unhealthy slum conditions and congestion include diarrhoea outbreaks for long periods of time which leads to instances of malnutrition, poor health, and death among young children [10]. According to Haddad et al, the urban poor and the urban underfed have increased, and will surpass those in rural areas [11]. The authors projected that in the next 20 years, there would be an increase in the urban poor, and with it would be increased levels of malnutrition. Recent research in the Kenyan context shows that ill health and poor nutrition are more common characteristics of children living in urban areas than rural areas [8]. A study using data from 15 countries showed that the urban advantage is being eroded for young children [12]. This is attributed to deterioration in conditions in urban areas rather than an improvement in rural areas. The deterioration in urban areas is attributed to the rapid population growth therein which has surpassed the provision of sanitation and health services [12-14].

### Women’s education and child malnutrition

Research shows that there is a strong linkage between maternal education and children’s health. Children born to educated women suffer less from malnutrition which manifests as underweight, wasting and stunting in children. Maternal education has been associated with nutrition outcomes among children in studies in various settings including Jamaica [15]; Bolivia [16]; and Kenya [17,18]. However, the mechanisms that link mother’s education and child health in general are still not well understood. Glewwe (1999) highlights three links through which education may affect child health [19]. First, formal education of mothers directly transfers health knowledge to future mothers. Second, the literacy and numeracy skills that women acquire in school enhance their ability to recognise illness and seek treatment for their children. Additionally, they are better able to read medical instructions for treatment of childhood illness and apply the treatment. Third, increased number of years in school makes women more receptive to modern medicine. Other studies have found a strong link between maternal education, social economic status and child nutritional status. This is because educated women are more likely to get steadier, higher paying jobs; to get married to men with higher education and higher income; and to live in better neighbourhoods, which have influence on child health and survival [16,20,21]. Studies have also found an association between maternal education and maternal depression [22], while maternal depression has been associated with poor child health outcomes, including poor nutritional outcomes [23]. Against this background, this study focuses on the urban poor in Kenya, a country that still registers a relatively high child mortality rate in Africa (74/1000 in 2008–09) [24]. Progress towards the 2015 Millennium Development Goals (MDG) targets has been slower than anticipated, the reason why there is need for further research into ways in which maternal education influences child nutrition. The purpose of this study is to better understand factors that influence poor health outcomes in the urban slums of Nairobi, Kenya. We seek to answer the question: Does mother’s education affect child nutritional status in the context of urban poverty?

## Methods

### Study setting

The study was carried out in two urban informal settlements of Korogocho and Viwandani in Nairobi, Kenya. The study was nested within the Nairobi Urban Health and Demographic Surveillance System (NUHDSS) run by the African Population and Health Research Center (APHRC). The NUHDSS follows a population of slightly more than 60,000 people; 57 % and 43 % from Viwandani and Korogocho slum respectively. The follow up comprises of a systematic quarterly recording of vital demographic events including births, deaths and migrations occurring among residents of all households in Korogocho and Viwandani since 2003. Other data that is collected includes: household assets, morbidity, and education which is also collected and updated regularly. The two slum areas are densely populated, and characterized by high unemployment rates, lack of a basic infrastructure, poor housing, violence, insecurity, and poor health indicators [8,25]. The two slums are heterogeneous particularly with regards to socio-economic status: Viwandani has relatively higher levels of education and employment, high mobility of residents and split households while the population in Korogocho is more stable with greater co-residence of spouses.

### Data source and data collection

This paper draws on data from the maternal and child health component of a broader longitudinal study entitled Urbanization, Poverty and Health Dynamics (UPHD) in sub-Saharan Africa, nested within the NUHDSS. The UPHD project addresses key health consequences of rapid urbanization and growing urban poverty at different stages of the life course namely childhood, adolescence, adulthood, and old age. Initially, all women who had given birth from September 2006 to January 2007, as well as their children were enrolled in the study starting in February 2007. Data were collected at baseline and updated every 4 months, at which point more mother and child pairs were enrolled. Anthropometric measurements for each child were taken during every update. Length for children aged less than 24 months was measured using a measuring board (Shorr Productions, LLC, Maryland, US) while height for those aged 24 months or more was measured using a height board (Seca 213, Seca GmbH, Germany). During each visit field interviewers administered questionnaires to collect data on vaccination, health care seeking and child care practices. In this study we utilize cross-sectional data of child stunting collected in the last round of data collection conducted between October 2009 and January 2010. The study involves eight sub-cohorts of children (n = 5156) aged 0–42 months enrolled between February 2007 and January 2010.

### Ethical considerations

The Urbanization, Poverty and Health Dynamics study was approved by the Ethical Review Board of the Kenya Medical Research Institute (KEMRI). The field workers were trained in research ethics and obtained informed consent from all respondents. The NUHDSS has also been approved by KEMRI’s Ethical Review Board. Verbal consent is routinely obtained from all the NUHDSS respondents.

### Variables and measurement

The analysis focuses on the effect of mother’s education on child stunting. The dependent variable is stunting; measured by height-for-age z-scores (HAZ). The height for age z-scores were generated using the World Health Organization (WHO) 2006 growth standards with the WHO Anthro 2005 program, Beta Version (World Health Organization, 2006). The variable was coded as “1” (stunted) if HAZ was < −2, and “0” (not stunted) if HAZ was ≥ −2. Mother’s education is the main predictor variable. The two education categories (primary education or less and secondary or higher) were coded as “1” and “0” respectively. The covariates included: child level characteristics, maternal demographic characteristics, maternal health knowledge and seeking behaviour; household characteristics and community characteristics.

#### *Child level characteristics*

Birth weight was constructed from the children’s weight reported on the birth card or mother's recall. Birth weight has two categories—less than 2500 g and above 2500 g. Gender of child has two categories; female and male.

#### *Maternal demographic characteristics*

Mother’s age has five categories: 15–19, 20–24, 25–29, 30–34, and 35+. The mother’s marital status was coded into 3 groups: currently in union (currently married or living together), ever in union (separated, divorced, and widowed), and never married. Parity defined as the number of children per woman was divided into three categories: first birth, second birth, and third birth and above. Ethnicity comprised of five categories: Kikuyu, Luhya, Luo, Kamba and others.

#### *Maternal health knowledge and seeking behaviour*

Pregnancy intentions, used as a proxy for family planning intention was categorized as wanted now, wanted later and not at all. Number of ante-natal visits had two categories; 4 or more visits and less than 4 visits. This measure of four ante-natal visits and above is what is recommended in Kenya. Place of delivery was categorized into three categories; health facility, Traditional birth attendants (TBA/home) and others (e.g. enroute to health facility). Knowledge on when to initiate complementary foods was categorized as “yes” (coded as “0”) if the mother knew that complementary foods should be initiated at 6 months as recommended by WHO, and “no” (coded as “1”) if the mother said she did not know, or if she said that the foods should be initiated earlier or later than 6 months.

#### *Household characteristics*

Wealth index was constructed from household assets and amenities using principal component analysis [26]. The three wealth index categories were; low, middle, and highest.

#### *Community characteristics*

The study site was used as a proxy for community level characteristics. The variable site was coded as “0” for Viwandani and “1” for Korogocho.

### Analytical techniques

The analysis was restricted to the most recent update for each child. Cross tabulation and univariable analysis was used to explore the relationship between mothers’ education and child stunting as well as the effect of other covariates. Cross tabulations were used to show the distribution of the various characteristics listed above among the stunted children. In addition, univariable analysis was done in an exploratory manner to determine the effect of each factor on child stunting. The univariable models were estimated using the binomial logistic regression.

Thereafter, we conducted a multiple logistic analyses. In the multiple logistic analyses we entered all the variables that were used in the univariable analysis. However, the explanatory variables were entered sequentially in groups. The key predictor (mother’s education) was entered into the model first; followed by child characteristics, maternal demographic characteristics, maternal health knowledge and healthcare seeking behavior, household characteristics and community level factors. We adjusted for clustering at the household level, to account for the nature of the data where some mothers had more than one child. Some explanatory variables had missing values for example child birth weight (34 %), maternal education (~ 18 %), ante-natal visits (~ 5 %), and social-economic status (~ 10 %). Imputation was done to address the missing values. We note that the household economic status was collected longitudinally in 2006, 2007, 2008 and 2009. In order to impute the missing data for social- economic status variable, we first used interpolation method to fill in the gap by computing the mean value for the existing data and substituting it for the missing household SES value. For the remaining missing data (from households that did not have SES information at any time-point (about 10 %), we used regression based imputation method to fill in the gaps.

Specifically, to generate the missing values for the variable with missing data, we used the following STATA commands to perform multiple imputation: mi impute chained (regress) for the continuous variables like child weight (34 % missing), mother’s age (about 17 % missing) and household SES level (before categorizing SES scores into three levels); mi impute logit for the categorical variables like mother’s education level (18 % missing) and the number of ante-natal visits (about 5 % missing); and mi impute mlogit for multi-category variables like ethnicity (16 % missing). The three multiple imputation methods are implemented in STATA version 12 based on different approaches used to perform multiple imputation for continuous and categorical variables [27-30]. Note, the implementation of the three set of multiple imputation commands require prior execution of the following two commands mi set and mi register which prepares and informs STATA to initialize and perform multiple imputation.

We also tested for the effects of imputation by generating a binary (0/1) variable representing unimputed/imputed data and including it into the model. The coefficient for the variable was not significant, an indication that the imputation process did not change the results.

## Results

### Descriptive statistics for child stunting

Table 1 presents percentages of stunted children by different explanatory variables. The results show that 44 % of boys are stunted compared to 34 % girls. Korogocho slum has slightly more stunted children with about 41 % compared to Viwandani with 38 %. Stunting is more common among children born to single mothers and older mothers, with 45 % and 46 % respectively. Mothers that have at most primary level of education have 43 % of their children stunted compared to 37 % for mothers with at least secondary level of education. About 35 % of children born to mothers who wanted to get pregnant at the time of conception were stunted compared to 44 % of children born to mothers who wanted the pregnancy later, and 42 % of children born to mothers who did not want to get pregnant at all. Overall, 71 % of children were born in a health facility while 26 % were born at home. Among those that were born in health facilities, 37 % were stunted compared to 45 % and 35 % who were born at home/TBA and en route to hospital respectively. Mothers with three and more births had 43 % of their children stunted compared to 41 % of mothers with two births, and 35 % among mothers with one birth. 62 % of children who had low birth weight (less than 2500) were stunted compared to 36 % of the children who were of optimal weight (above 2500).

**Table 1:** Characteristics of children aged between 0–42 months in the study

**Factor**	**Category**	**Stunted (%)**	**Number of Subjects**	**%**
**Child’s Sex**	Male	44.2	2410	50.5
	Female	34.2	2360	49.5
**Slum**	Korogocho	40.9	2490	52.2
	Viwandani	37.5	2280	47.8
**Mother’s Education level**	Primary and below	43.1	2,961	76.7
	Secondary +	36.9	897	23.3
**Mother's age**	15-19	39.9	406	10.3
	20-24	40.1	1415	35.9
	25-29	42.2	1185	30.1
	30-34	42.9	564	14.3
	35 >	45.6	373	9.5
**Mother’s Marital Status**	Currently in union	37.8	3954	82.9
	Ever in union	48.3	375	7.9
	Never married	44.8	440	9.2
**Pregnancy Intentions**	Wanted now (Ref)	35.2	2378	50.0
	Wanted later	43.5	1773	37.3
	Not at all	42.3	608	12.8
**Ante-natal Visits**	Less than 4	38.7	2460	54.9
	4 or more	38.5	2018	45.1
**Place of delivery**	Health Facility	37.4	3390	71.2
	TBA & Home	44.6	1239	26.0
	Others(enroute to hospital)	34.9	132	2.8
**Mother’s Parity**	1 birth	34.5	1633	34.3
	2 births	40.6	1325	27.8
	3 and above	42.5	1806	37.9
**Child’s birth weight**	Less than 2500	61.8	220	7.0
	Above 2500	36.2	2940	93.0
**Knowledge when to introduce other foods**	No	41.3	1477	31.0
	Yes	38.4	3293	69.0
**Social Economic Status**	Lowest	42.5	812	27.3
	Middle	44.5	991	33.3
	Highest	39.7	1172	39.4
**Mother’s Ethnicity**	Kikuyu	43.2	1057	26.7
	Luhya	39	689	17.4
	Luo	41.5	768	19.4
	Kamba	42.5	835	21.1
	Others	40.6	606	15.3

The univariable results presented in Table 2 show that mothers education, child gender, birth weight, place of birth, slum site, mothers’ marital status, pregnancy intentions and social economic status can significantly determine a child’s nutritional status (p < 0.05). Additionally, being the first born, a female of normal weight, born in a health facility, by a mother who is currently in union, who has at least secondary education, in a household that has high SES, will be protective against child malnutrition (p < 0.05). Moreover, children born in Korogocho have a high tendency to be stunted compared to children born to mothers who live in Viwandani. However, mothers’ age, number of ante-natal visits and ethnicity do not determine stunting.

**Table 2:** Odds ratios showing the association between mothers’ education and child stunting based on univariable analysis

**Factor**	**Category**	**Odds ratio**	**95 % Conf. Interval**	**p-values**
**Child’s Sex**	Female (ref.)	-		
	Male	1.520	(1.353 1.710)	0.000
**Slum**	Viwandani (ref.)			
	Korogocho	1.157	(1.029 1.299)	0.014
**Mother’s Education**	Secondary + (ref.)	-		
	Primary and below	1.281	(1.096 1.498)	0.002
**Mother's age**	15-19 (ref.)	-		
	20-24	1.010	(0.806 1.265)	0.931
	25-29	1.099	(0.874 1.383)	0.419
	30-34	1.132	(0.873 1.467)	0.349
	35 >	1.261	(0.949 1.677)	0.11
**Mother’s Marital status**	Currently in union (ref.)	-		
	Ever in union	1.536	(1.242 1.988)	0.000
	Never married	1.335	(1.094 1.628)	0.004
**Pregnancy Intentions**	Wanted now (ref.)	-		
	Wanted later	1.420	(1.252 1.611)	0.000
	Not at all	1.348	(1.124 1.616)	0.001
**Ante natal Visits**	Above 4 (ref.)	-		
	Less than 4	1.008	(0.893 1.138)	0.894
**Place of delivery**	Health Facility (ref.)	-		
	TBA & Home	1.343	(1.177 1.532)	0.000
	Others	0.894	(0.621 1.287)	0.547
**Mother’s Parity**	One birth (ref.)	-		
	2 births	1.299	(1.118 1.509)	0.001
	3 and above	1.406	(1.225 1.615)	0.000
**Child’s birth weight**	Above 2500 (ref.)	-		
	Less than 2500	2.848	(2.148 3.777)	0.000
**Knowledge when to introduce other foods**	Yes (ref.)	-		
	No	1.131	(0.999 1.281)	0.054
**Social Economic Status**	Lowest (ref.)	-		
	Middle	1.022	(0.924 1.131)	0.674
	Highest	0.843	(0.765 0.928)	0.000
**Mother’s Ethnicity**	Kikuyu (ref.)	-		
	Luhya	0.938	(0.845 1.041)	0.229
	Luo	0.927	(0.837 1.026)	0.144
	Kamba	0.996	(0.901 1.100)	0.934
	Others	0.818	(0.735 0.910)	0.000

### Multiple logistic regressions results (Effect of mother’s education on child stunting)

Table 3 presents the multiple regression results predicting the effect of mother’s education on child stunting in the urban slums of Nairobi. Six models were fitted sequentially in this estimation, with an aim of observing the impact of mother’s education on child’s stunting and whether the impact of education is sustained when other variables (mothers’ and child characteristics, household, and community level) are introduced into the model. The estimation started with a simple model—mothers’ education regressed against child stunting. Thereafter, we sequentially added other grouped variables. Model 1 is the baseline model which introduces one explanatory variable, mothers’ education, model 2 introduces the child characteristics, while model 3 adds mother’s demographic characteristics. Model 4 adds variables related to the mothers’ health knowledge and health seeking behaviours. Model 5 and 6 add household and community characteristics respectively.

**Table 3:** Multivariable analysis of the association between mothers’ education and child stunting – with imputed data

**Factor**	**Model 1 Coef. (95 % CI)**	**p-val**	**Model 2 Coef. (95 % CI)**	**p-val**	**Model 3 Coef. (95 % CI)**	**p-val**
**Mother Education**						
Secondary + (ref.)	-		-		-	
Primary and below	1.29 (1.11, 1.51)	0.001	1.30 (1.11, 1.52)	0.001	1.26 (1.08, 1.48)	0.004
**Child’s Sex**						
Female (ref.)	-		-		-	
Male			1.57 (1.40, 1.77)	0.000	1.57 (1.40, 1.77)	0.000
**Child’s Birth Weight**						
Above 2500 (ref.)	-		-		-	
Less than 2500			2.80 (2.12, 3.70)	0.000	2.72 (2.06 , 3.60)	0.000
**Mother's Age**						
15-19 (ref.)	-		-		-	
20-24					1.03 (0.83, 1.27)	0.818
25-29					1.04 (0.82, 1.33)	0.734
30-34					1.04 (0.78, 1.39)	0.787
35 >					1.13 (0.82, 1.56)	0.471
**Mother’s Marital Status**						
Curr. in union (ref.)	-		-		-	
Ever in union					1.47 (1.18, 1.84)	0.001
Never married					1.56 (1.25, 1.94)	0.000
**Mother’s Parity**						
First birth (ref.)	-		-		-	
2 births					1.31 (1.11, 1.55)	0.002
3 and above					1.39 (1.14, 1.69)	0.001
**Mother’s Ethnicity**						
Kikuyu (ref.)	-		-		-	
Luhya					0.91 (0.75, 1.11)	0.350
Luo					0.88 (0.73, 1.05)	0.162
Kamba					1.03 (0.85, 1.23)	0.781
Others					1.01 (0.44, 2.30)	0.978
*Number of observations*	*4770*		*4770*		*4770*	
*Log-Likelihood*	*−3165.2*		*−3105.7*		*−3080.69*	
**Factor**	**Model 4 Coef. (95 % CI)**	**p-val**	**Model 5 Coef. (95 % CI)**	**p-val**	**Model 6 Coef. (95 % CI)**	**p-val**
**Mother’s Education**						
Secondary + (ref.)	-		-		-	
Primary and below	1.24 (1.05, 1.46)	0.010	1.22 (1.03,1.43)	0.018	1.21 (1.02,1.43)	0.025
**Child’s Sex**						
Female (ref.)	-		-		-	
Male	1.59 (1.41, 1.80)	0.000	1.60 (1.42,1.80)	0.000	1.60 (1.42,1.80)	0.000
**Child’s Birth Weight**						
Above 2500 (ref.)	-		-		-	
Less than 2500	2.99 (2.25, 3.97)	0.000	2.96 (2.23,3.93)	0.000	2.96 (2.23,3.93)	0.000
**Mother's Age**						
15-19 (ref.)	-		-		-	
20-24	1.04 (0.84, 1.29)	0.689	1.05 (0.85,1.30)	0.655	1.05 (0.85,1.30)	0.642
25-29	1.10 (0.86, 1.41)	0.426	1.12 (0.88,1.43)	0.357	1.12 (0.88,1.44)	0.349
30-34	1.11 (0.83, 1.48)	0.494	1.12 (0.84,1.51)	0.437	1.12 (0.84,1.51)	0.432
35 >	1.20 (0.87, 1.67)	0.270	0.21 (0.87,1.68)	0.258	1.21 (0.87,1.68)	0.254
**Mother’s Marital status**						
Currently in union (ref.)	-		-		-	
Ever in union	1.41 (1.13, 1.77)	0.003	1.37 (1.09,1.72)	0.007	1.36 (1.09,1.71)	0.007
Never married	1.64 (1.14, 1.78)	0.002	1.35 (1.08,1.70)	0.009	1.35 (1.08,1.69)	0.010
**Mother’s Parity**						
First birth (ref.)						
2 births	1.23 (1.04, 1.47)	0.016	1.21 (1.02,1.44)	0.028	1.21 (1.02,1.44)	0.028
3 and above	1.22 (1.00, 1.49)	0.054	1.16 (0.95,1.43)	0.151	1.16 (0.94,1.42)	0.156
**Mother’s Ethnicity**						
Kikuyu (ref.)	-		-		-	
Luhya	0.83 (0.68, 1.01)	0.063	0.82 (0.67,1.00)	0.047	0.74 (0.67,1.00)	0.047
Luo	0.78 (0.65, 0.95)	0.013	0.77 (0.64,0.94)	0.008	0.77 (0.64,0.93)	0.008
Kamba	0.97 (0.80, 1.17)	0.743	0.98 (0.81,1.18)	0.822	0.99 (0.82,1.20)	0.924
Others	0.97 (0.42, 2.21)	0.937	0.99 (0.43, 2.26)	0.975	0.99 (0.43,2.26)	0.980
**Pregnancy Intentions**						
Wanted now (ref.)	-		-		-	
Wanted later	1.38 (1.20, 1.58)	0.000	1.37 (1.19,1.57)	0.000	1.36 (1.19,1.56)	0.000
Not ever wanted	1.19 (0.96, 1.44)	0.113	1.17 (0.95,1.43)	0.132	1.16 (0.95,1.42)	0.157
**Ante-natal Visits**						
Above 4 (ref.)	-		-		-	
Less than 4	0.95 (0.84, 1.08)	0.454	0.95 (0.83,1.07)	0.398	0.95 (0.83,1.07)	0.384
**Place of Delivery**						
Health Facility (ref.)						
TBA & Home	1.39 (1.20, 1.61)	0.000	1.38 (1.19, 1.60)	0.000	1.39 (1.20,1.61)	0.000
Others (enroute to hospital)	0.98 (0.67, 1.42)	0.903	1.01 (0.69,1.47)	0.958	1.02 (0.70,1.50)	0.905
**Knowledge when to introduce other foods**						
Yes (ref.)	-		-		-	
No	1.07 (0.94, 1.22)	0.299	1.07 (0.94,1.22)	0.324	1.07 (0.94,1.22)	0.317
**Social Economic Status**						
Lowest (ref.)	-		-		-	
Middle			0.93 (0.80,1.07)	0.312	0.93 (0.80,1.08)	0.333
Highest			0.77 (0.66,0.91)	0.001	0.78 (0.66,0.66)	0.002
**Slum of Residence**						
Korogocho (ref.)	-		-		-	
Viwandani					0.96 (0.84,1.11)	0.590
*Number of observations*	*4770*		*4770*		*4770*	
*Log-Likelihood*	*−3055.98*		*−3050.38*		*−3050.2*	

Model 1 indicates that child stunting is significantly related to the mothers’ education level (p < 0.01). The odds of child stunting are 29 % higher for mothers with no education or lower than secondary education, relative to mothers that have at least secondary education. Addition of child characteristics (child birth weight and gender) in model 2, do not change the significance of the relationship between mothers’ education and child stunting. However, both child birth weight and gender are strongly related to stunting (p < 0.01, respectively). Males have 57 % higher probability of being stunted compared to girls, while children of low birth weight are close to three times more likely to be stunted relative to children of normal birth weight.

Model 3 introduces mothers’ demographic characteristics (mothers’ age, marital status, parity and ethnicity). The relationship between mother’s education and child stunting was only slightly attenuated by the presence of these demographic characteristics. However, mothers’ marital status, parity, and ethnicity are independently associated to child stunting. The odds of stunting for children born to mothers who were never married are 56 % higher relative to those who are currently in union respectively (p < 0.05). Mother’s parity was also marginally associated with stunting. The odds of stunting for children born to mothers who have two births, and three or more births are 31 % and 39 % higher compared to those that have one child respectively (p < 0.05). Model 4 controlling for both mother’s health knowledge and health-related behavior (family planning intentions, ante-natal visits, place of delivery and knowledge on when weaning should be initiated) had little effect on the relationship between maternal education and child stunting. However, the timing of pregnancy (whether child was wanted at conception or later or not wanted at all) and place of delivery independently determine the nutritional status of a child. The odds of stunting for a child born to a mother who gives birth in a TBA facility or at home are 39 % higher compared to giving birth in a health facility (P < 0.05). Those children whose mothers wanted to get them later had 38 % higher odds of being stunted compared to those children whose mothers wanted them at conception (P < 0.05). The association between knowledge on when to initiate complementary feeding and child stunting was not significant (p > 0.1).

Inclusion of the household socioeconomic status (SES) in model 5 marginally reduces the significance of the relationship between mothers’ education and child stunting. However, being in middle SES category does not have significant effects on child’s stunting but being in highest SES category reduces the chances of stunting among children. The introduction of SES reduces the education effect on child stunting from 1.24 to 1.22, which is still significant at 5 % significance level. Consequently, controlling for household socioeconomic status and other demographic characteristics, results in the odds for child stunting being 22 % higher for mothers who have no education or lower than secondary level education relative to those who have at least secondary education level. Introduction of slum of residence in model 6 leads to a reductionon the effects of mother’s education from 22 % to 21 %, but mother’s education is still significantly associated to child stunting. Slum of residence is not significantly related to child stunting. Separate models were run for community level variable(s), (Korogocho and Viwandani). The results gave almost the same effects and none of the model had significant effects of education on stunting. In addition, the interaction term between education and slum site for the combined data was not significant.

It is important to note that most covariates had high level of correlation. To account for the correlation, we computed the significance of the correlation among the variables. Results indicate that the correlation levels among covariates are high and significant (significance level below 0.05) except correlations between the sex of the child and other covariates. The observed high correlation among covariates has effects on the fitted logistic regression models. For example, at univariate levels the site (Viwandania and Korogocho) was significantly related to stunting but at multivariate level, the variable (site) was no longer significantly related to stunting. This can be explained by high correlation that exist between the site and other variables like marital education level and age, place of delivery and social economic status of a household.

## Discussion and conclusions

The objective of this study was to assess the effect of maternal education on child nutritional status in the two slums of Nairobi. The prevalence of stunting among children aged up to 42 months in these communities was close to 40 %. We found that mother’s education is an important predictor for child stunting. Compared to other studies [16,19,20], we found that the effect of mother’s education on child stunting is only minimally attenuated by factors at child, maternal, household and community level. Therefore, we find a persisting significance of mother’s education on child’s stunting in this context.

Child level factors including sex and birth weight are independently and strongly associated with stunting. While the association between birth weight and nutritional status has been well documented and understood in sub-Saharan Africa [31,32], few studies have documented gender difference with regards to malnutrition in young children particularly in sub-Saharan Africa [17,33]. It has been argued that such differences occur in low socio-economic status settings [34]. Despite the strong significance of these child level factors in influencing child stunting, our study concludes that they do not substantially alter the effect of education on child stunting.

In addition to child level factors, maternal level factors including marital status, parity, ethnicity and mothers’ health knowledge and health related behaviour including pregnancy intentions used as a proxy for family planning, and place of delivery used as a proxy for health seeking behaviour were significantly related to child stunting. The relationship between maternal level factors and child nutritional status has also been documented in several other studies [16]. These factors however only minimally attenuate the effect of mother’s education on stunting.

The introduction of household wealth index into the model also minimally attenuates the effect of education on stunting, which somehow differs with findings from others settings [16,20]. Moreover, we find that SES is also significantly related to child stunting similar to findings from other studies [16,34], which found a statistically significant relationship between SES and child malnutrition. We also find that the introduction of slum residence marginally reduces the magnitude of the effect of mother’s education on stunting.

Our results were limited by the absence of direct measures for family planning, SES, and community level characteristics for which proxy measures were used. In the case of SES, an index was constructed from household assets. The use of proxy measures and the construction of an index might have affected the impact of these variables on the dependent variable. Education status, the main predictor variable of interest was collected as a categorical variable. This may reduce the level of precision in measurement of the effect. Overall, our study has significant policy implications for child health in urban slums in Kenya. Evidence indicates that improving nutritional status has many benefits that cannot be overemphasized including improved health and survival, cognitive development and future human capital [5,6,35]. Our study has indicated that mother’s education is an important predictor for child stunting. This suggests that improving mother’s years of schooling may have significant influence on child nutritional status and ultimately alter the poverty cycle as stunting is a key predictor of human capital. Other factors influencing child nutritional status in the slums of Nairobi include child level factors (sex and birth weight), maternal level factors (marital status, parity, ethnicity, health knowledge and health seeking behaviour) and SES.

Inclusion of health knowledge skills in school curricula may lead to substantial improvement in child nutritional status by directly enabling the girls who are future mothers to have an improved health knowledge, practices, and health seeking behavior [19]. This study indicates that among children born in the slums, a substantial proportion was born at home or with the assistance of a TBA. This therefore indicates that many mothers do not adequately benefit from the baby-friendly hospital initiative being implemented in Kenya. The aim of the initiative is to enhance optimal breastfeeding and young child feeding practices, thus improving infant and child nutritional status [36]. The study therefore calls for awareness creation to enhance delivery at health facilities. Policies targeted at improving livelihoods of the urban poor may result in reduced malnutrition and ultimately alter the poverty cycle.

## Competing interests

The authors have no competing interests financial or otherwise, arising from the publication of this article.

## Authors’ contributions

BAA: Conceptualization of the idea, writing of the manuscript, finalization of manuscript in readiness for submission. JMC: Data analysis, writing of the manuscript and approval for submission. EWK-M: Design of the study, project management, writing of the manuscript and approval for submission. All authors read and approved the final manuscript.

## Authors information

Benta A. Abuya is an Associate Research Scientist with the African Population and Health Research Center (APHRC), in Nairobi, Kenya. Her research interests include comparative education, education policy issues of access, equity and quality, and the linkages between education and health.

James Ciera is a Research/Knowledge translation scientist at the African Institute for Development Policy (AFIDEP) and formerly a Post-Doctoral Fellow at APHRC. James has a PhD is Statistical Sciences from University of Padova (Italy) and an Msc in Applied Statistics and Biostatistics from LimburgsUniversitair Centrum (Belgium). His research interests are in application of longitudinal data analysis and Bayesian statistics in health and education relatedproblems.

Elizabeth Wambui Kimani-Murage is an Associate Research Scientist with the African Population and Health Research Center and a Wellcome Trust Fellow. Her current research interests include maternal and child health issues, malnutrition including obesity and food security.

## Pre-publication history

The pre-publication history for this paper can be accessed here:

http://www.biomedcentral.com/1471-2431/12/80/prepub
